# Self-Oscillations of Submerged Liquid Crystal Elastomer Beams Driven by Light and Self-Shadowing

**DOI:** 10.1007/s10659-024-10091-8

**Published:** 2024-10-09

**Authors:** Reza Norouzikudiani, Luciano Teresi, Antonio DeSimone

**Affiliations:** 1 BioRobotics Institute, Scuola Superiore Sant’Anna, Viale Rinaldo Piaggio 34, Pontedera, Italy; 2 Dipartimento di Ingegneria Industriale, Elettronica e Meccanica, University Roma Tre, via della Vasca Navale 84, Rome, Italy; 3 SISSA-Scuola Internazionale Superiore di Studi Avanzati, Trieste, Italy

**Keywords:** Liquid crystal elastomer, Fluid structure interaction, Self-oscillation, Self-shadowing, 74, 74F05, 74F10, 7410

## Abstract

Liquid Crystal Elastomers (LCEs) are responsive materials that undergo significant, reversible deformations when exposed to external stimuli such as light, heat, and humidity. Light actuation, in particular, offers versatile control over LCE properties, enabling complex deformations. A notable phenomenon in LCEs is self-oscillation under constant illumination. Understanding the physics underlying this dynamic response, and especially the role of interactions with a surrounding fluid medium, is still crucial for optimizing the performance of LCEs. In this study, we have developed a multi-physics fluid-structure interaction model to explore the self-oscillation phenomenon of immersed LCE beams exposed to light. We consider a beam clamped at one end, originally vertical, and exposed to horizontal light rays of constant intensity focused near the fixed edge. Illumination causes the beam to bend towards the light due to a temperature gradient. As the free end of the beam surpasses the horizontal line through the clamp, self-shadowing induces cooling, initiating the self-oscillation phenomenon. The negative feedback resulting from self-shadowing injects energy into the system, with sustained self-oscillations in spite of the energy dissipation in the surrounding fluid. Our investigation involves parametric studies exploring the impact of beam length and light intensity on the amplitude, frequency, and mode of oscillation. Our findings indicate that the self-oscillation initiates above a certain critical light intensity, which is length-dependent. Also, shorter lengths induce oscillations in the beam with the first mode of vibration, while increasing the length changes the elasticity property of the beam and triggers the second mode. Additionally, applying higher light intensity may trigger composite complex modes, while the frequency of oscillation increases with the intensity of the light if the mode of oscillation remains constant.

## Introduction

In recent years, soft robotics has emerged as a promising field to create machines which are capable of performing various tasks in different conditions while adapting to changes of environmental conditions [1]. This interesting field promises to revolutionize various application fields, ranging from energy harvesting in soft materials to artificial muscles and even particle transport mechanisms in microfluidics by mimicking cilia-like motions [2, 3]. Various types of soft materials including hydrogels, shape memory polymers (SMPs), magneto-rhelogical elastomers (MREs), and liquid crystal elatomers (LCEs) have been explored in soft robotic systems and applications. Among them, LCEs stand out for their unique properties and versatile capabilities.

LCEs represent a class of responsive materials that exhibit both the elasticity property of polymers and the spontaneous ordering property of liquid crystals [4, 5]. They can switch between a nematic phase with aligned directors and an isotropic phase with no director alignment, depending on their order parameter tensor. The order parameter can be altered and controlled by changing the temperature ($T$) (relative to the transition temperature $T_{ni}$) or by applying external stimuli such as light, heat, magnetic field, electric field and humidity [6, 7], which results in significant, spontaneous and reversible macroscopic deformations.

Depending on their geometry, director pattern alignment, and nature of the external stimuli, LCEs can undergo various modes of deformation such as stretching, bending, and torsion [8, 9], providing engineers with a wide range of possibilities for designing soft robotic systems tailored to specific tasks and environments. One particularly fascinating phenomenon observed in LCEs is self-oscillation under constant light actuation inspired by biological rhythms such as heartbeat and leaves oscillations [10]. This phenomenon showcases the dynamic nature of LCEs and their potential for bio-mimetic applications in soft robotics and biomedical engineering.

Various experimental studies have been carried out to achieve self-oscillation in LCE beams surrounded by air and subjected to constant light, either due to the self-shadowing effect [11, 12], or the alternating actuation of top and bottom surfaces [13]. Moreover, recent experimental work has explored the generation of self-oscillation in a planar LCE beam immersed in water due to the self-shadowing effect [14]. Unlike previous studies on self-oscillation of LCE beams, this research presented a novel approach to generate non-reciprocal self-oscillation in LCE beams by utilizing two orthogonal laser beams. The induced non-reciprocity opens up the opportunity to use LCEs in bio-mimetic applications such as the translocation of micro-particles by liquid propulsion, through a mechanism that replicates muco-ciliary clearance. A light-powered micro-swimmer made of four beams stemming from a common central point, bio-inspired by the anatomy and motion of ephyras, has been discussed in [15]. In all these studies, photo-thermal effects, photo-chemical effects or a combination of both was responsible for the deformation and bending of the beams.

Light actuation of LCE beams involves several physical phenomena and scientific domains such as structural mechanics, heat transfer, light absorption, chemical reactions, and, in case of actuation in fluid, fluid mechanics. Furthermore, the interaction between these physics becomes more complex when dealing with large self-oscillation, due to the nonlinearity of the governing equations. Therefore, it is crucial to develop mathematical and numerical models to gain a deeper understanding of the underlying physics. This is indeed necessary to characterize which deformations and functionalities can be achieved, and at which actuation cost. Specifically, for self-oscillation, numerical models can help us to study the amplitude, frequency, and mode of oscillation under different conditions.

Several numerical and theoretical studies have investigated the light actuation of LCE beams to explore and predict their quasi-static [16–22] and dynamic [23–26] responses, particularly focusing on the self-oscillation phenomenon. In these studies, the generated self-oscillations were usually obtained through chemical effects by assuming a small relaxation value for the cis molecules. However, in reality, the relaxation time of cis molecules is much higher [27], making it unreasonable to generate self-sustained oscillation due to the saturation of the fraction of cis molecules [25]. Moreover, self-oscillations in previous works were obtained by alternating the actuation of the top and bottom surfaces of the LCE beams, while in most experimental studies, the self-shadowing effect under constant illumination was the main cause for the occurrence of self-oscillation. Additionally, prior studies on the self-oscillation of LCE beams mostly developed their models based on the linear beam theory considering only local damping, which is insufficient for accurately modeling the effects of surrounding fluid when deformations are large. Therefore, there is still an urgent need for developing more efficient and general multi-physics models based on large deformation theories that consider the self-shadowing effect and fluid dynamic physics to simulate problems such as self-oscillations of a submerged LCE beam subjected to constant illumination.

In this paper, we develop a 2D multi-physics fluid structure interaction (FSI) model to investigate the large self-oscillations of an immersed photo-responsive LCE beam, which has not been addressed in previous numerical studies. The model is then implemented using COMSOL multiphysics software, and it is used to conduct numerical parametric studies to explore the role of various parameters in determining the observed physical response of the system.

In Sect. 2, we describe the multi-physics model, considering the nonlinear coupling between structural mechanics, fluid mechanics and heat transfer, and we utilize the Beer-Lambert equation to model light absorption. In our model, deformation and bending are caused by thermal effects under applied light, particularly the temperature gradient across the thickness. Moreover, unlike previous modeling studies on the self-oscillations of LCE beam under illumination, we consider explicitly in the model the self-shadowing effect, namely, the possibility that the deformed beam may interfere with light rays coming from a far away light source by projecting a shadow onto itself. In Sect. 3, the self oscillation of a submerged LCE beam under constant applied illumination is simulated using the developed model. We then conduct several parametric studies to explore the effect of different parameters, such as light intensity and beam length on amplitude, mode and frequency of the oscillation and to shed light on the physics of this phenomenon. The obtained results are compared to experiment [14], and the similarities and discrepancies between the simulation and experimental results are highlighted and discussed.

## Multi-Physics Modeling

**Fig. 1:**
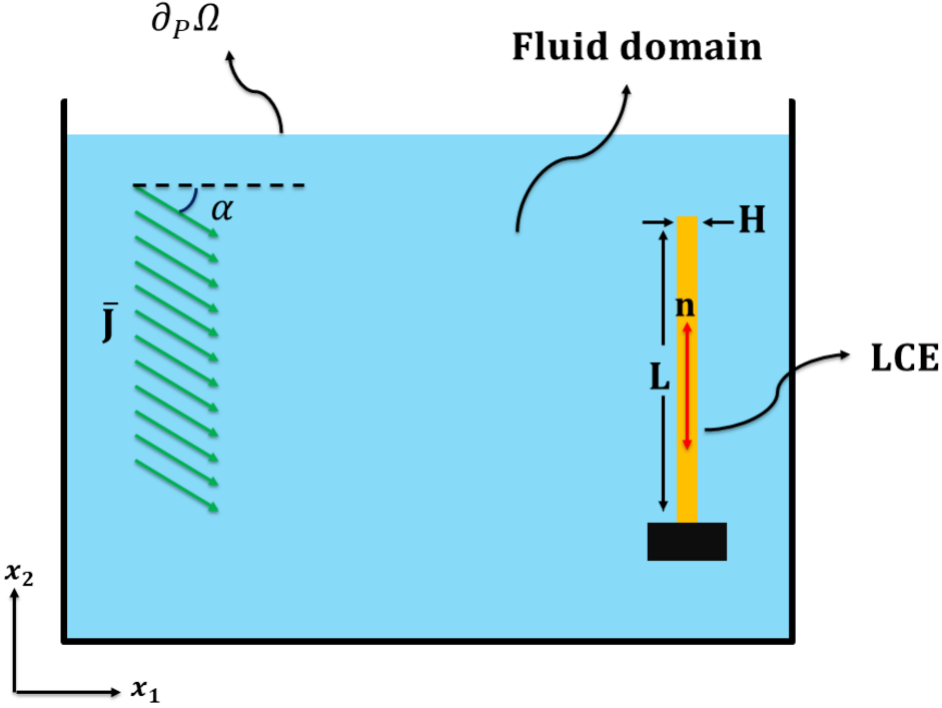
A thin photoactive nematic LCE beam (right, yellow) subjected to illumination from a light source (left, green) and immersed in water (cyan). Cartoon not to scale

Our multi-physics model involves fluid-solid interactions, heat transfer, and light-induced heating under a radiation source. We consider a thin photoactive LCE beam, surrounded by a fluid and exposed to a light radiation coming from a source at infinity so that it can be modeled as a plane wave coming from a given constant direction. The LCE beam in its reference configuration has a vertical straight shape, is clamped at the bottom edge, and is in the nematic state with directors aligned parallel to the long axis, Fig. 1.

**Fig. 2:**
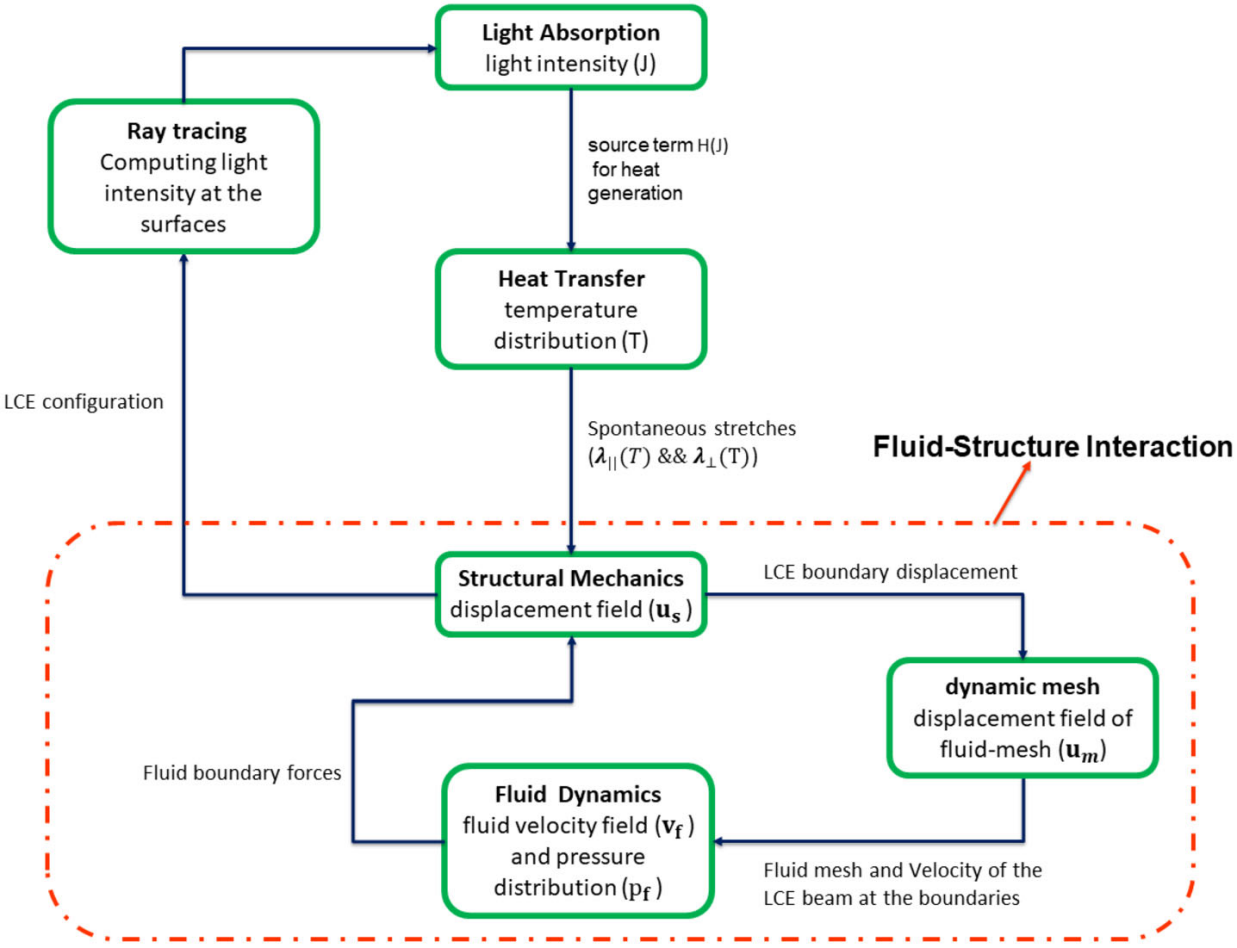
Modeling diagram for actuation of immersed LCE beams under illumination consisting all involved physics

Under light exposure, the temperature of the beam increases and yields a nematic-to-isotropic transition of the LCE; since the temperature change is higher on the side exposed to radiation, the beam bends towards this side. Upon bending, the effects of light radiation vary thus yielding a coupling between the beam configuration and its temperature. Figure 2 contains a schematic view of the coupling between the various physics involved in our model.

We implement all physics in a 2D geometry, assuming plane strains for solid mechanics; we define a square domain $\Omega =D\times D$, containing the body domain $\Omega _{b}= H\times L$ and the moving mesh domain $\Omega _{m}=\Omega -\Omega _{b}$. The body domain $\Omega _{b}$ represents the (vertical) reference configuration of a thin photoactive LCE beam having length L, thickness H, and width W; $\Omega _{m}$ represents the initial configuration of the fluid domain $\Omega _{f}$ surrounding the solid, see Fig. 1 (not in scale). Fluid mechanics is solved in $\Omega _{f}$ by using the moving mesh approach.

### Beam Motion

The displacement of the beam $\mathbf{u}_{s}=\mathbf{u}_{s}(\mathbf{X},t)$, with $(\mathbf{X},t) \in \Omega _{b}\times T$ is determined by the balance of linear momentum:

1 $$ \begin{aligned} \rho _{s} \ddot{\mathbf{u}}_{s} & = \nabla \cdot \mathbf{S}, \textrm{ on }\Omega _{b}\times T, \quad \textrm{balance of linear momentum,} \\ \mathbf{S}\,\mathbf{n}&= \mathbf{t}_{f}, \textrm{ on }\partial _{f} \Omega _{b}\times T, \quad \textrm{boundary load,} \\ \mathbf{u}&= 0, \textrm{ on }\partial _{u}\Omega _{b}\times T, \quad \textrm{constraint,} \\ \mathbf{u}_{s}(\mathbf{X},0)&=0, \textrm{ on }\Omega _{b}\times \{0\}, \quad \textrm{initial condition,} \end{aligned} $$

where $\rho _{s}$ is the beam density, $\mathbf{S}$ the first Piola-Kirchhoff stress, $\mathbf{t}_{f}$ the force coming from the surrounding fluid; moreover $\partial _{u}\Omega _{b}$ is the clamped bottom edge, and $\partial _{f}\Omega _{b}$ are the remaining three edges where the beam interacts with the fluid. At time $t$, the current position $\mathbf{x}$ of a point $\mathbf{X}$ of the beam, and the current configuration $\Omega _{bt}$ are given by

2 $$ \begin{aligned} \mathbf{x}=\mathbf{X}+\mathbf{u}_{s}(\mathbf{X},t), \quad \Omega _{bt}= \{\mathbf{x}=\mathbf{X}+\mathbf{u}_{s}(\mathbf{X},t),\, \mathbf{X} \in \Omega _{b}\}. \end{aligned} $$

The spontaneous strain associated with the nematic-to-isotropic transition is described by the strain tensor $\mathbf{F}_{s}$, defined by

3 $$ \begin{aligned} \begin{aligned} \mathbf{F}_{s}=\lambda _{||}\,\mathbf{n}\otimes \mathbf{n}+\lambda _{\perp}\,(\mathbf{I}-\mathbf{n}\otimes \mathbf{n}), \quad \text{ with }\mathbf{n}\text{ the nematic director.} \end{aligned} \end{aligned} $$

$\mathbf{F}_{s}$ rewrites in components as $(F_{s})_{ij}=\lambda _{||} n_{i}n_{j}+\lambda _{\perp} (\delta _{ij}-n_{i}n_{j})$, where $\delta _{ij}$ is the Kronecker delta. The nematic stretches $\lambda _{||}$ and $\lambda _{\perp}$ are related by the request $\operatorname{det}(\mathbf{F}_{s})=1$, which implies $\lambda _{||}\,(\lambda _{\perp})^{2}=1$. The relation between the nematic stretch $\lambda _{||}$ and the temperature $T$ is modeled with the experimental law

4 $$ \begin{aligned} \lambda _{||}= \lambda _{||}(T) = 1 + \epsilon (T). \end{aligned} $$

It follows that a temperature variation yields spontaneous deformations in both parallel ($\lambda _{||}$), and perpendicular ($\lambda _{ \perp}$) directions to the LC’s director $\mathbf{n}$. Given (3), the elastic deformation $\mathbf{F}_{e}$ is measured with respect to the spontaneous one by the formula

5 $$ \begin{aligned} \mathbf{F}_{e}= \mathbf{F}\, \mathbf{F}_{s}^{-1}, \quad \textrm{ with } \mathbf{F}=\mathbf{I}+\nabla \mathbf{u}_{s}. \end{aligned} $$

We assume that the elastic energy of the beam is governed by the incompressible Neo-Hookean model:

6 $$ \begin{aligned} W= \frac{\mu}{2}\, (\mathbf{C}_{e}\cdot \mathbf{I}-3)-p\,(\operatorname{det}( \mathbf{F}_{e})-1), \end{aligned} $$

where $\mathbf{C}_{e}\cdot \mathbf{I}=\operatorname{tr}(\mathbf{C}_{e})$ is the first invariant of the elastic right Cauchy-Green strain $\mathbf{C}_{e}=\mathbf{F}_{e}^{T}\,\mathbf{F}_{e}$, $\mu $ is the shear modulus, and $p$ is the Lagrange multiplier enforcing the volumetric constraint $\operatorname{det}(\mathbf{F}_{e})=1$. Eventually, the stress $\mathbf{S}$ is defined as the derivative of the elastic energy $W$ with respect to the deformation gradient $\mathbf{F}$:

7 $$ \begin{aligned} \mathbf{S}= \frac{\partial W}{\partial \mathbf{F}} = \mu \,J_{s} \mathbf{F}\,\mathbf{C}_{s}^{-1} -p\,\mathbf{F}^{*}, \end{aligned} $$

where $\mathbf{F}^{*}=\operatorname{det}(\mathbf{F})\,\mathbf{F}^{-T}$ is the cofactor of $\mathbf{F}$, and $J_{s}=\operatorname{det}\mathbf{F}_{s}$, $\mathbf{C}_{s}=\mathbf{F}_{s}^{T}\,\mathbf{F}_{s}$.

### Light Absorption

To formulate the light absorption equation in the reference configuration, we made certain simplifications and approximations. In our setting, light comes from the left vertical edge of $\Omega $, is incident on the sample surface and is attenuated exponentially within the sample along a path $s(\mathbf{x},t)$ with characteristic penetration depth $d_{r}$. We consider the pull-back of the attenuation path in the reference configuration as being aligned with the $\mathbf{X}_{1}$-direction, which is normal to the surface in the reference configuration. Since $d_{r}$ is typically very small, the errors accompanying this approximation will be small. A more precise description would require solving the light propagation problem both outside (as we do, see below) and inside (as done, e.g., in [17]) the domain $\Omega _{j}(t)$ occupied by the beam.

Considering the assumptions described above, the light intensity in the beam is described by the state variable $J=J(\mathbf{X},t)$, with $(\mathbf{X},t) \in \Omega _{b}\times T$. Following [28], we use the one-dimensional Beer-Lambert model for light absorption:

8 $$ \begin{aligned} \begin{aligned} \frac{\partial J}{\partial X_{1}} &= -\frac{J}{d_{r}}, \textrm{ on }\Omega _{b}\times T, \quad \textrm{Beer-Lambert law,} \\ J &= J_{0}, \textrm{ on }\partial \Omega _{j}(t)\times T, \quad \textrm{boundary intensity,} \\ J&=0, \textrm{ on }\Omega _{b}\times \{0\}, \quad \textrm{initial intensity,} \end{aligned} \end{aligned} $$

where $d_{r}$ is the penetration depth and $\partial \Omega _{j}(t)$ is portion of the beam boundary that is illuminated during the motion. In our experimental setting, $\partial \Omega _{j}(t)$ is always a subset of the edge that in the reference configuration is vertical and faces the light source, see Fig. 1. If no obstacle is present between the light source and the beam boundary, then $J_{0}$ can be obtained from the intensity of incoming light $\bar{J}$, and the angle between the incoming light direction $\mathbf{n}_{l}$ and the normal to the current boundary of the beam $\mathbf{n}_{b}$:

9 $$ \begin{aligned} J_{0}= -\bar{J} \mathbf{n}_{l}\cdot \mathbf{n}_{b} = \bar{J} \cos ( \theta ), \,\textrm{ for } \pi /2 < \theta < 3\,\pi /2,\quad J_{0}= 0, \,\textrm{ otherwise.} \end{aligned} $$

where $\bar{J}$ is given by

10 $$ \begin{aligned} \bar{J} = J_{o}\, \exp \left (- \frac{(x_{2}-x_{2}^{o})^{2}}{2\,\sigma ^{2}}\right )), \quad [J_{o}]=W/m^{2}. \end{aligned} $$

**Fig. 3:**
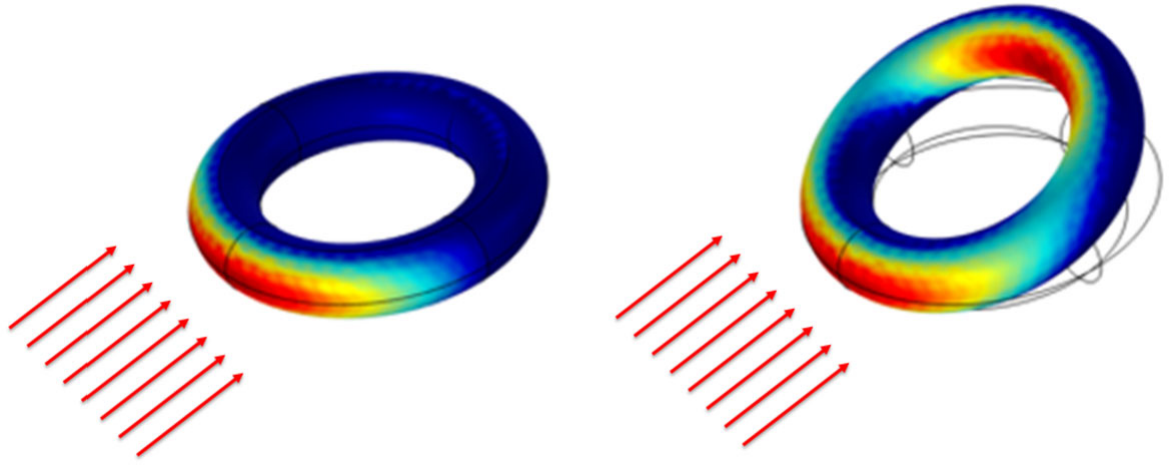
Test of self-shadowing on a deformable 3D torus. Left: in the initial configuration only a portion of the surface is exposed to light. Right: in the deformed configuration, light illuminates a larger portion of the boundary, including a part in the inner side of the torus that was previously shadowed by the front part

The above equation represents the incoming light with a Gaussian profile along the vertical direction $x_{2}$, centered at $x_{2}^{o}$ and having a variance of $\sigma $. Additionally, $J_{o}$ denotes the peak amplitude of the incoming light at the center. As inferred, illumination with large variance can results in an almost uniform illumination with the intensity of $\bar{J} = J_{o}$ as shown in Fig. 1. Equation (9) is the special case which has been considered in previous theoretical studies [16, 20, 25]. More generally, however, parts of an illuminated object may project a shadow on other parts: this is the phenomenon of self-shadowing. At the points that are left in the dark by self shadows, the incident light intensity vanishes instead of being given by (9). The region not hit by light because of self-shadowing can be determined by using the equations for geometric optics in the domain $\Omega \setminus \Omega _{j}(t)$ that are solved by the module for heat radiation of COMSOL multiphysics. We have tested extensively the possibility of modeling correctly self-shadowing in complex geometries, such as the illumination of a 3D torus from a horizontal light coming from a far away source, see Fig. 3. We remark that since the current configuration of the body is an unknown of the problem and regions separated by large distances may project shadows one onto another, illumination in the presence of self-shadowing yields a non-local, nonlinear coupling between heat transfer and elasticity. By contrast, equation (9) is a local boundary condition for the beam thermoelasticity problem.

### Heat Transfer

The temperature of the beam $T=T(\mathbf{X},t)$, with $(\mathbf{X},t) \in \Omega _{b}\times T$ is determined by the heat transfer equation:

11 $$ \begin{aligned} \begin{aligned} \rho _{s}\, C_{s}\,\dot{T} &= \nabla \cdot \mathbf{q}+ Q, \textrm{ on }\Omega _{b}\times T, \quad \textrm{balance of heat power}, \\ -\mathbf{q}\cdot \mathbf{n}&= q_{s}, \textrm{ on }\partial \Omega _{b} \times T, \quad \textrm{boundary heat source}, \\ T&=T_{o}, \textrm{ on }\Omega _{b}\times \{0\}, \quad \textrm{initial temperature}, \end{aligned} \end{aligned} $$

with $Q$ a bulk source of heat power generated by light absorption, and where $\rho _{s}$, $C_{s}$, and $\mathbf{K}_{s}$ are the density, the heat capacity and the thermal conductivity of the LCE beam, respectively; the heat flux $\mathbf{q}$ is given by

12 $$ \begin{aligned} \mathbf{q}= -\mathbf{K}_{s}\,\nabla T. \end{aligned} $$

We note that for the heat transfer problem (11) we assign a boundary source $q_{s}$ on the whole boundary $\partial \Omega _{b}$, and thus we do not control the boundary temperature (no Dirichelet conditions). The heat boundary source $q_{s}$ represents a convective flux given by

13 $$ \begin{aligned} q_{s}= h\,(T_{env}-T), \end{aligned} $$

where $h$ is the heat transfer coefficient, and $T_{env}$ is the fluid temperature, which is assumed constant. Finally, the bulk heat source $Q$ can also be expressed as the divergence of the Poynting flux [28].

14 $$ \begin{aligned} Q(\mathbf{X},t) = -\frac{\partial J}{\partial X_{1}}(\mathbf{X},t), \quad [Q]=W/m^{3}. \end{aligned} $$

### Fluid Dynamics

The motion of the fluid $\mathbf{v}=\mathbf{v}(\mathbf{x},t)$, with $(\mathbf{x},t) \in \Omega _{f}\times T$ is described by the Navier-Stokes equations for an incompressible Newtonian flow:

15 $$ \begin{aligned} \rho _{f}(\dot {\mathbf{v}}+\nabla \mathbf{v}\, \mathbf{v}) &= \nabla \cdot (\eta \,\operatorname{sym}\nabla \mathbf{v}-p_{f}\, \mathbf{I}) +\mathbf{f}_{f} \textrm{ on }\Omega _{f}\times T, \, \textrm{balance of forces,} \\ \mathbf{v}&= 0, \textrm{ on }\partial _{N}\Omega \times T, \quad \textrm{no-slip wall condition}, \\ \mathbf{v}&= \mathbf{v}_{s}, \textrm{ on }\partial _{s}\Omega _{b} \times T, \quad \textrm{fluid-solid interface}, \\ \mathbf{v}&= 0, \textrm{ on }\Omega _{f}\times \{0\}, \quad \textrm{initial condition on velocity}, \\ p_{f} &= 0, \textrm{ on }\Omega _{f}\times \{0\}, \quad \textrm{initial condition on pressure}, \\ p_{f} &= 0, \textrm{ on } \partial _{p}\Omega \times T, \quad \textrm{pressure boundary condition}, \\ \rho _{f}\,\nabla \cdot \mathbf{v}&=0, \textrm{ on } \Omega _{f} \times T, \quad \textrm{incompressibility constraint}. \end{aligned} $$

Here, $\rho _{f}$, $\eta $, and $p_{f}$ are the density, the dynamic viscosity, and the pressure of the fluid, respectively; $\mathbf{f}_{f}$ is the bulk force on the fluid. The boundary $\partial \Omega $ has two parts: $\partial \Omega =\partial _{p}\Omega \cup \partial _{N}\Omega $. The section $\partial _{p}\Omega $ represents the top surface where the pressure $p_{f}=0$ and no back-flow condition is applied. This is the condition that approximates most effectively the laboratory conditions in the experiments we are trying to model, where there is an essentially stationary free-surface far away from the region occupied by the beam, and which do not require the full resolution of the motion of the free surface. Our choice is more accurate that assuming the presence of a no-slip wall, and is much simpler than modeling the presence of a free surface in detail. The rest of the boundary $\partial _{N}\Omega $ is instead made by a no-slip wall, where we assume $\mathbf{v}=0$.

We note that $\partial \Omega $ is the boundary of the square domain considered as an impermeable tank, where we have a wall condition; $\partial _{s}\Omega _{b}\times T$ is the interface fluid-solid, where we require that the fluid velocity $\mathbf{v}$ be equal to the spatial field of the solid velocity $\mathbf{v}_{s}$ defined as

16 $$ \begin{aligned} \mathbf{v}_{s}(\mathbf{x},t) =\dot{\mathbf{u}}_{s}(\mathbf{X},t), \quad \textrm{ for } \mathbf{x}=\mathbf{X}+\mathbf{u}_{s}(\mathbf{X},t). \end{aligned} $$

The forces $\mathbf{t}_{f}$ exerted by the fluid on the LCE beam boundaries are evaluated by

17 $$ \begin{aligned} \mathbf{t}_{f} =-\left (\eta \,\operatorname{sym}\nabla \mathbf{v}-p_{f}\, \mathbf{I}\right )\,\mathbf{n}_{b}. \end{aligned} $$

### Moving Mesh

To track the motion of the fluid domain $\Omega _{f}$ we use the moving mesh approach. The displacement of the moving domain $\mathbf{u}_{m}=\mathbf{u}_{m}(\mathbf{X},t)$, with $(\mathbf{X},t)\in \Omega _{m}\times T$ is determined by a Yeoh smoothing-mesh model. Thus, the current position $\mathbf{x}$ of a point of the mesh domain $\Omega _{m}$ and the current position of the fluid domain $\Omega _{f}$ are given by

18 $$ \begin{aligned} \mathbf{x}= \mathbf{X}+\mathbf{u}_{m}(\mathbf{X},t), \quad \Omega _{f}= \{ \mathbf{x}= \mathbf{X}+\mathbf{u}_{m}(\mathbf{X},t), \mathbf{X} \in \Omega _{m}\}. \end{aligned} $$

All the differential operator that appear in (15), such as time derivative, gradient and divergence, are computed accounting for the motion of $\Omega _{f}$ with respect to $\Omega _{d}$.

### Numerical Implementation

The fluid-structure interaction (FSI) problem is implemented in COMSOL software through its built-in interfaces. COMSOL handles the FSI problem using an Arbitrary Lagrangian-Eulerian (ALE) formulation. In this method, Navier-Stokes equations are solved on a freely moving deformed mesh ($\mathbf{x}_{m}$) which constitutes the fluid domain. The deformation of the moving mesh is computed based on the movement of the LCE beam’s boundaries and the smoothing Yeoh method. The Yeoh method, inspired by hyperelastic materials, seeks to minimize the mesh deformation energy, described by the strain energy, $W_{m}$:

19 $$ W_{m}=\frac{1}{2} \int _{\Omega _{m}} C_{1}\left (I_{1}^{m}-3\right )+C_{2} \left (I_{1}^{m}-3\right )^{2}+C_{3}\left (I_{1}^{m}-3\right )^{3}+ \kappa (J^{m}-1)^{2} d V. $$

In the above equation, $C_{1}$, $C_{2}$, $C_{3}$ are artificial material constants and $\kappa $ is the artificial bulk modulus, and $J^{m}$ and $I^{m}_{1}$ are invariants. By default, $C_{1}$ and $C_{3}$ are set to 1 and 0, respectively, while $C_{2}$, which controls the nonlinear stiffening of the artificial material under deformation, is set to 100 in this problem.

Moreover, the implicit adaptive step-size Generalized Alpha (GA) solver is used for the time-stepping. A quasi-Newton algorithm is employed to iteratively solve the non-linear algebraic system resulting from the finite element discretization at each time step. The direct solver MUMPS is chosen for the solution of the linearized system at each iteration.

## Results and Discussions

In this section, we use the developed multi-physics model to investigate the bending and oscillation of LCE beams immersed in water and subjected to light. As mentioned, the induced spontaneous deformations caused by variation of temperature lead to macroscopic deformations such as stretching and bending in LCE beam under illumination. The variation of the spontaneous stretch parallel to the LC’s alignment ($\lambda _{||}$) as function of temperature is extracted from experimental data provided by [14]. To simplify the problem and reduce the computational cost, the deformation of the beam in $X_{3}$-direction is neglected and only in-plane deformation is investigated. Consistently with this and with the assumption of incompressibility, we take

20 $$ \begin{aligned} \lambda _{\perp} = \frac{1}{\lambda _{||}}\,. \end{aligned} $$

Since the LCE beam is in the nematic phase in the reference configuration, as the temperature rises under illumination, it contracts along the alignment direction, causing $\lambda _{||}$ to decrease to a value less than 1. Also, due to the incompressibility of the LCE beam, this contraction ($\lambda _{||}$) results in an expansion perpendicular to the alignment direction, causing $\lambda _{\perp }$ to increase to a value greater than 1. It is also assumed that the surrounding fluid is governed by unsteady Stokes flow. As a consequence, the Navier-Stokes equation (15) is simplified by neglecting the convection term on the left-hand side:

21 $$ \begin{aligned} \rho _{f}\dot{\mathbf{v}}= \nabla \cdot (\eta \,\operatorname{sym}\nabla \mathbf{v}-p_{f}\,\mathbf{I}) +\mathbf{f}_{f}. \end{aligned} $$

**Fig. 4:**
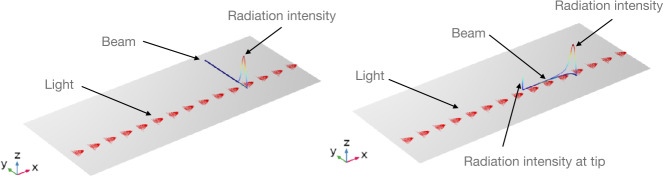
Light intensity profile at beam boundary under illumination. Left) At the initial configuration the beam (dark blue) is orthogonal to the light having a Gaussian profile (red arrows); the light intensity is represented by the line plot above the beam. Right) During oscillation, the beam is bent towards the coming light and the light intensity on its boundary has a different profile; note that also the tip of the beam is illuminated

Inspired by [14], the coupled model is solved to investigate the self-oscillations of a submerged LCE beam subjected to constant lighting, and due to self-shadowing effects. To replicate the experiment, it is assumed that the LCE beam is exposed locally to constant-intensity light near the fixed edge at an angle $\alpha =0$, and characterizing by a Gaussian profile with $x^{o}_{2}=8.5\text{ mm}$, and $\sigma =0.265\text{ mm}$., see (10) and Fig. 4. For these simulations, the value of the material and geometric properties of the LCE beam and the surrounding fluid are also provided in Table 1.

**Table 1:** Material and geometric properties of the LCE beam and water

Symbol	Definition	Value	Unit
*D*	Domain Edge Length	25 × 10^−3^	*m*
*W*	Width	1 × 10^−3^	*m*
*H*	Thickness	60 × 10^−6^	*m*
*E*	Young’s modulus	5	*MPa*
$\rho _{s}$	LCE mass density	1400	$Kg/m^{3}$
$d_{r}$	Penetration depth	5 × 10^−6^	*m*
$C_{s}$	Specific heat	1600	*J*/(*kg*.*K*)
$k_{||}$	LCE heat conduction coefficient	0.8	*W*/(*m*.*K*)
$k_{\perp}$	LCE heat conduction coefficient	0.2	*W*/(*m*.*K*)
*h*	Water heat convection coefficient	900	$W/m^{2}K$
$T_{env}$	Environment temperature	293.15	*K*
$T_{0}$	Initial temperature	293.15	*K*
$\rho _{f}$	Water mass density	1000	$Kg/m^{3}$
*η*	Dynamic viscosity of water	1 × 10^−3^	*Pa*.*s*

**Fig. 5:**
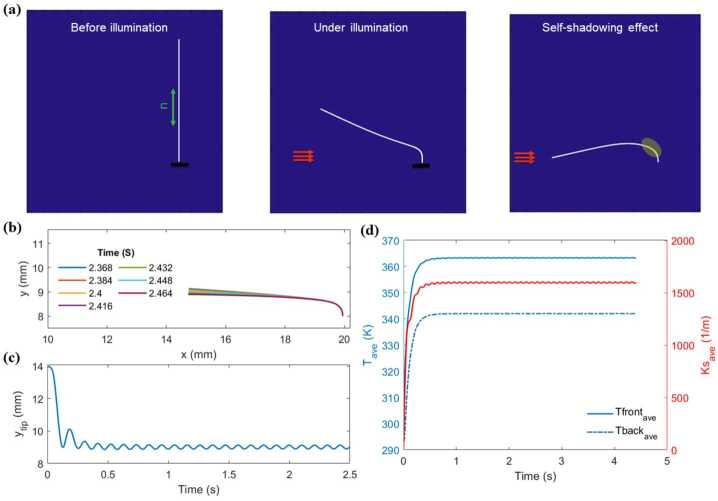
a) Bending deformation of an immersed LCE beam exposed locally to constant-light near the fixed end, and the resultant self-shadowing effect. b) Snapshots of the self-oscillation, and c) vertical displacement of the tip of the LCE beam with $L=6\text{ mm}$ subjected to constant Gaussian light intensity with the peak value of $J_{o}=18{,}000~\frac{\text{mW}}{\text{cm}^{2}}$. d) Time evolution of the average temperatures of the front and back surfaces of the LCE beam (left axis), and averaged spontaneous curvature (right axis)

As shown in Fig. 5a, when the LCE beam is illuminated, it bends towards the light source due to temperature gradient. As it passes the horizontal position due to its inertia, a self-shadowing effect occurs, leading to cooling of previously exposed parts of the beam and initiating self-oscillations.

Figures 5b and 5c show snapshots and vertical tip displacement of a beam with $L=6\text{ mm}$ subjected to a constant Gaussian light intensity with a peak amplitude of $J_{o}=18{,}000~\frac{\text{mW}}{\text{cm}^{2}}$. These figures reveal that the beam oscillates with the first mode of vibration of a cantilever at a frequency of 9.5 Hz. Moreover, to get deeper insight into the bending and oscillation behavior of the beam, average temperatures of the front and back surfaces of the LCE beam are plotted on the left axis of the Fig. 5d. Only the regions near the fixed end are considered to compute the averages temperatures. As shown, under illumination, temperatures of both front and back surfaces increase and due to the gradient of temperature the beam bends towards the light source. However, after some time, average temperatures begin to oscillate around equilibrium values due to the self-shadowing effect, resulting in the oscillation of the temperature gradient and self-oscillation of the LCE beam.

Further insight can be obtained from the spontaneous curvature $k_{s}$ defined as

22 $$ \begin{aligned} k_{s}=\frac{1}{I_{m}} \int _{\mathrm{A}}-\mathrm{X_{1}} \varepsilon _{ \mathrm{s}}(T) \mathrm{d} A, \end{aligned} $$

and used in [25] to explore the bending and self-oscillation of an LCE beam. In the above equation, $\varepsilon _{s}=\lambda _{||}-1$ is the parallel spontaneous strain, $I_{m}$ is the second moment of the inertia, and $A$ is the area of the cross section. This feature, computed by considering the temperature profile along the thickness, provides more information than only using the temperatures of the front and back surfaces alone. The average spontaneous curvature is also computed for the regions near the fixed end and plotted on the right axis of the Fig. 5d. As shown, under illumination, the spontaneous curvature increases, causing the LCE beam to bend towards the light and then oscillate around its equilibrium position due to the oscillation of the spontaneous curvature.

**Fig. 6:**
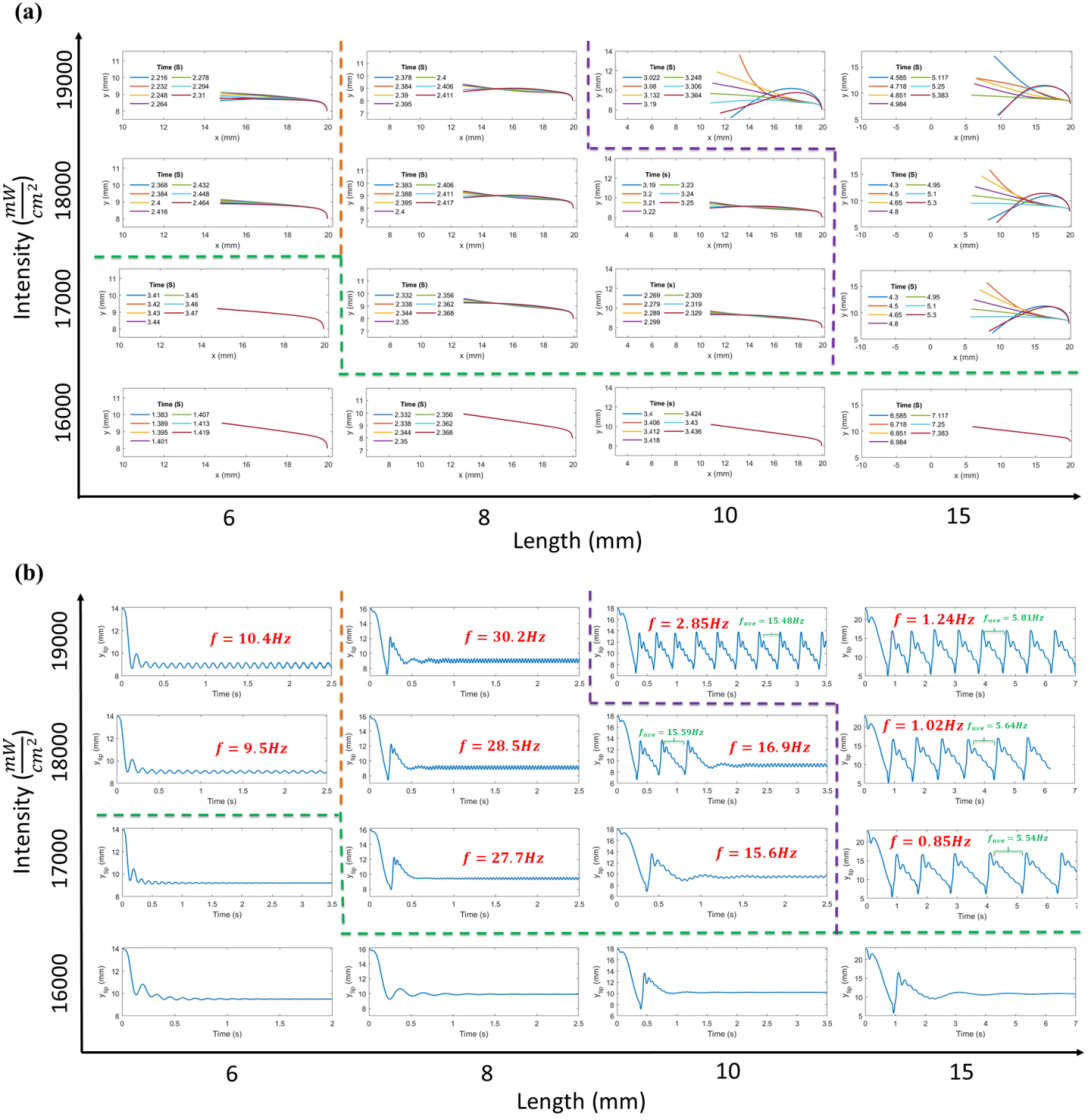
Parametric studies for self-oscillation of immersed LCE beams under illumination. For each pairs of length and light intensity, the snapshots of beam’s configuration (a) and the time evolution of tip vertical displacement (b) are plotted

Parametric studies are also conducted using the developed model for various lengths and light intensities. In Fig. 6, snapshots of beam’s configuration at different times and the evolution of the vertical displacement of the tip are plotted for each pairs of beam length and light intensity. As illustrated, self-oscillation initiates above a certain light intensity for all lengths. The threshold values are approximately similar for different lengths, with the exception of the beam with the length of 6 mm, which shows slight variation. Below these thresholds, the beam bends towards the light source under illumination, and oscillates around its equilibrium position with a decaying amplitude, eventually stopping due to absence of the self shadowing effect and dissipation by the surrounding fluid. However, above these thresholds, the LCE beam starts to oscillate as the self-shadowing causes cooling in the region near fixed edge, inducing variations in the temperature gradient, which introduces additional energy into the system in each cycle and compensates for fluid dissipation, enabling sustained oscillations.

Moreover, according to the Fig. 6, beams with shorter lengths like L = 6 mm self-oscillate with the first mode of vibration under all explored light intensities above the critical one. Increasing the length from 6 mm to the 8 mm alters the elasticity property of the LCE beam, causing it to self-oscillate with the second mode of the vibration. Despite this increase in length, the frequency of the oscillation rises due to the change in the oscillation mode. Conversely, extending the beam length from 8 mm to 10 mm decreases the frequency of the oscillation as the mode of the vibration remains constant, except at a high light intensity with peak amplitude of $19{,}000~\frac{\text{mW}}{\text{cm}^{2}}$, which induces a complex oscillation. This complex oscillation is also observed in the results for the length of 15 mm. Two distinct frequencies are identified in these oscillations: the lower one (red) corresponds to oscillations with large and constant amplitude, while the higher one (green) represents the averaged frequency for the oscillation with low and decaying amplitude during the bending deformation of the LCE beam towards the light. Finally, it can be inferred from Fig. 6 that increasing the light intensity at each specific length leads to a rise in the oscillation’s frequency if the mode of the oscillation remains unchanged.

**Fig. 7:**
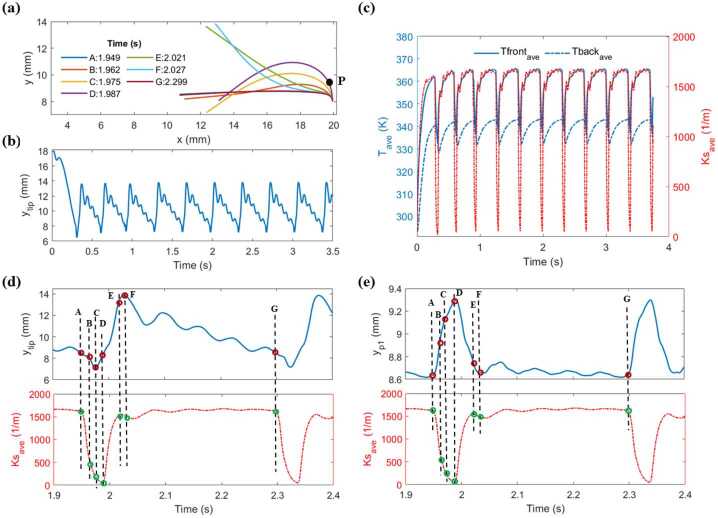
Self-oscillation of an immersed LCE beam length of 10 mm subjected to light with the peak amplitude of $19{,}000~\frac{\text{mW}}{\text{cm}^{2}}$. a) Snapshots of beam’s positions at different times (A-G). b) Tip vertical displacement over time. c) Time evolution of the averaged temperatures (left axis) and spontaneous curvature (right axis). d) Time evolution of the tip vertical displacement (top) and averaged spontaneous curvature (bottom). e) Time evolution of the displacement of the point $P$ (top) and averaged spontaneous curvature (bottom)

As mentioned earlier, increasing the length and light intensity to larger values can lead to self-oscillation with complex deformation. To gain better understanding of this phenomenon, the oscillation behavior of the beam with length of 10 mm subjected to light with the peak amplitude of $19{,}000~\frac{\text{mW}}{\text{cm}^{2}}$ is investigated in more detail in Fig. 7. Figure 7(a-c) depict the snapshots of beam’s position at different time steps (A to G), the time evolution of the tip vertical displacement, and the evolution of the average temperatures and spontaneous curvature over time, respectively. According to these figures, the beam bends towards the light source under illumination due to the rise in spontaneous curvature induced by temperature gradFient along the thickness. It then overshoots the equilibrium position because of its inertia and fully shadows the areas near the edges which results in the cooling of the sample and, in turn, in a significant reduction of the spontaneous curvature. Therefore, the beam bends back towards its initial position and generates oscillations with a large amplitude. For a more detailed analysis of the oscillation, the vertical displacement of the tip and point $P$ near the fixed edge (marked in Fig. 7a with black circle) for an specific period of the oscillation are plotted in Fig. 7d and Fig. 7e, respectively. The averaged spontaneous curvature is plotted on the right axis of these figures. Additionally, times A to G shown in Fig. 7a are marked on all the curves with red and green symbols. At time A, the beam is almost horizontally positioned (Fig. 7a) with highest spontaneous curvature (Fig. 7d) and moves downward. From time A to B, the area near the tip start to shadow the area near the fixed edge, cooling the sample and significantly reducing the averaged spontaneous curvature. This results in the back bending in the area near the fixed edge (Fig. 7e). From time B to C, despite the back bending in the area near the fixed edge, the tip part moves in the opposite direction because of the elasticity of the beam (Fig. 7d). Therefore, the tip part keeps shadowing the area near the fixed edge and further decreases the spontaneous curvature, causing the point P to still move upward (Fig. 7e). From time C to D, the tip part begins to bend back (Fig. 7d) and follows the movement of the area near the fixed edge (P). However, the area near the edge is still in shadow (Fig. 7a), resulting in more reduction in the spontaneous curvature. From time D to E, shadowing effects starts to diminish, leading to a sharp rise in spontaneous curvature. This sharp change in the spontaneous curvature bends the area near the edge (Fig. 7e) towards the horizontal position which causes the tip part to move in the opposite direction (Fig. 7d) due to the elasticity of the beam. From time E to F, spontaneous curvature decreases slightly as edge part becomes more horizontal and receives less light. From time F to G, the tip moves downward with a delay with respect to the edge movement, oscillating until it reaches a horizontal position at time G. Additionally, this oscillatory motion continually alters the angle between the light direction and the surface normal near the fixed edge, leading to oscillations in induced curvature until time G. To conclude, unlike point P, the dynamics of the tip does not only follow the variation of averaged spontaneous curvature because of the elasticity property of the beam. In other words, the dynamics of the tip is controlled by a combination of both the variation of the averaged spontaneous curvature, and of its elastic properties.

**Fig. 8:**
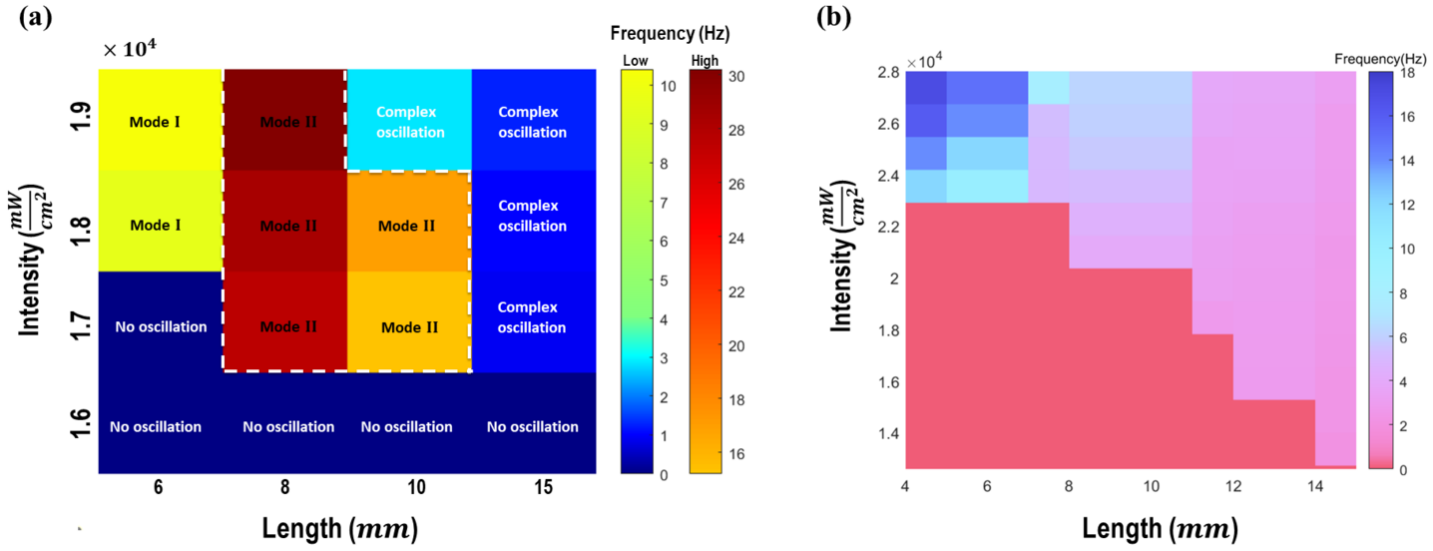
a) Color map summarizing the oscillation frequencies and mode shapes for different lengths under various applied light intensities. Two distinct color bars are utilized for better visualization. b) Color map for different light intensities and lengths reconstructed from experimental data in [14]

**Fig. 9:**
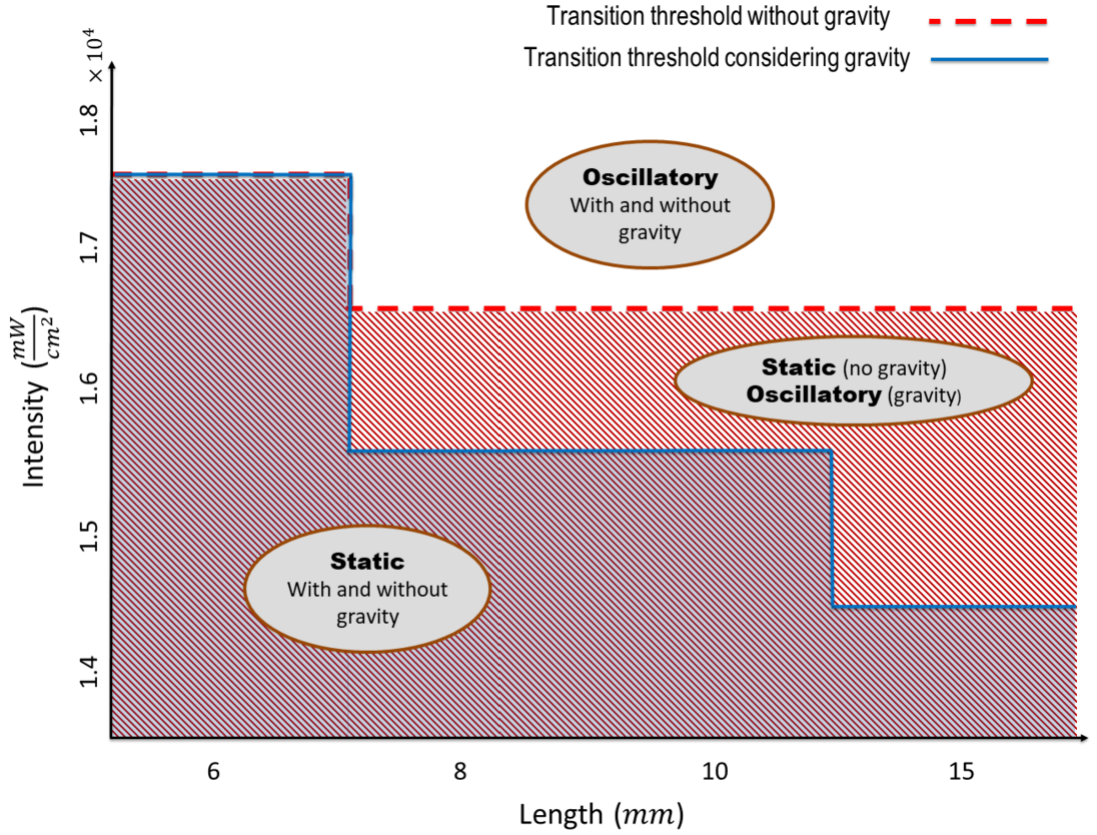
The impact of gravitational force on light intensity thresholds for transition from static to oscillatory motion across different lengths

To qualitatively compare the simulation with the experimental observations [14], the color map of the oscillation frequency for different lengths and light intensities are plotted in Fig. 8a. To better visualize the distinction between various simulation, two different color bars are used in the plot. Also, a similar color map is reconstructed using the experimental data provided in [14]. These figures indicate that for various length, there is a transition between static and oscillatory motion in both experiment and simulation when the light intensity exceeds a critical value. However, in the simulations, the line giving the dependence on beam length of the critical light intensity is relatively flat, whereas the experimental transition line is more jagged. To address this discrepancy, the effect of gravitational force is also investigated in our simulations. Figure 9 illustrates the variation in light intensity thresholds for the transition from static to oscillatory motion across different lengths, both with and without incorporating the gravity force in the model. As shown in this figure, accounting for the gravity force in the model decreases the light intensity thresholds for various lengths, where this reduction becomes more significant as the length increases. This adjustment makes the transition line between static and oscillatory motion more closely aligned with the experimental observations. Moreover, both experiment and simulation demonstrate that the oscillation frequency grows by increasing the applied light intensity at a fixed length. Furthermore, increasing the length leads to change of oscillation mode in the simulation, while in the experiment, the oscillation mode remains constant as the length increases. One possible reason for this discrepancy is that the heat transfer parameters used in the simulation, such as heat convection and conduction coefficients are not provided in the experimental study; instead, approximate values from the literature are used. Another reason might be the omission of the visco-elastic behavior of the LCE beam in the model which can affect the beam’s elasticity, dissipation and its oscillation mode. Neglecting the temperature dependence of the elastic modulus could also be a possible reason for the mismatch, which can be resolved by either considering the neoclassical model for LCEs [5] or modifying the current model by including the experimental data for the variation of Young’s modulus with temperature. Another issue worth of further investigation is the effect of retaining the convective term in equation (15).

## Conclusion

In this paper, we developed a 2D fluid-structure interaction (FSI) model to investigate the light actuation of LCE beams immersed in water. Our model incorporates various physical phenomena, including light absorption, heat diffusion, nonlinear elasticity of an active (i.e., stimulus-responsive) material, and fluid mechanics. We assumed that the bending deformation occurring in the LCE beam is only caused by photo-thermal effects, in particular the temperature gradient across the thickness of the beam. Using our model, we simulated the self-oscillation of immersed LCE beams due to the self-shadowing effect under constant illumination, which has not been previously modelled. The self-shadowing effect leads to cooling of the LCE beam, allowing additional energy to be injected into the system to overcome fluid dissipation, and resulting in sustained oscillations. Parametric studies were conducted to explore the effect of beam length and light intensity on self-oscillation behaviour.

Our results demonstrate the existence of a critical light intensity to trigger self-oscillations from a static state, and that further increasing the light intensity at a fixed length leads to an increase in the self-oscillations frequency. These observations align reasonably well with experimental observations from the literature.

Our simulations indicate also that the threshold light intensity to generate self-oscillations in the LCE beam is approximately constant for different lengths. However, experimental observations [14] show a more jagged transition threshold between static and oscillatory motion for different lengths. To address this mismatch, the effect of gravity was also investigated. The results demonstrated that the transition line between static and oscillatory motion become more similar to the experimental observations. Additionally, in simulations, shorter beams oscillate in the first mode, while increasing the length affects the elasticity of the beam and causes the beam to oscillate in the second mode. However, experimental observations have so far shown oscillations only in the first mode for all lengths. This discrepancy may be attributed to factors such as neglecting visco-elastic effects in the LCE beam and not precisely defining the values for heat transfer parameters such as convection and conduction coefficients. Shedding further light on these discrepancy may lead to valuable new insight in the mechanisms governing the physics of LCEs. In future work, we plan to address these issues by investigating the effects of heat transfer parameters, the viscoelasticity of the LCE beam, the temperature dependence of the elastic modulus using a neoclassical model for LCEs, and the inertial terms in fluid flow on the self-oscillation behavior. In conclusion, our study provides valuable insights into the self-oscillation of immersed LCE beams due to the self-shadowing effect and highlights areas for further investigation to improve the agreement between model-based theoretical predictions end experimental observations.

## Data Availability

No datasets were generated or analysed during the current study.
